# Sex differences in the association between major cardiovascular risk factors in midlife and dementia: a cohort study using data from the UK Biobank

**DOI:** 10.1186/s12916-021-01980-z

**Published:** 2021-05-19

**Authors:** Jessica Gong, Katie Harris, Sanne A. E. Peters, Mark Woodward

**Affiliations:** 1 The George Institute for Global Health, University of New South Wales, Level 5, 1 King St, Newtown, NSW 2042 Australia; 2 The George Institute for Global Health, Imperial College London, Central Working - Fourth Floor, Translation and Innovation Hub, Imperial College London, 80 Wood Lane, London, W12 0BZ UK; 3 Julius Center for Health Sciences and Primary Care, University Medical Center Utrecht, Utrecht University, PO Box 85500, 3508 GA Utrecht, The Netherlands; 4 Welch Center for Prevention, Epidemiology and Clinical Research, Johns Hopkins University, Baltimore, MD USA

**Keywords:** Sex difference, Risk factor, Dementia, UK Biobank

## Abstract

**Background:**

Sex differences in major cardiovascular risk factors for incident (fatal or non-fatal) all-cause dementia were assessed in the UK Biobank. The effects of these risk factors on all-cause dementia were explored by age and socioeconomic status (SES).

**Methods:**

Cox proportional hazards models were used to estimate hazard ratios (HRs) and women-to-men ratio of HRs (RHR) with 95% confidence intervals (CIs) for systolic blood pressure (SBP) and diastolic blood pressure (DBP), smoking, diabetes, adiposity, stroke, SES and lipids with dementia. Poisson regression was used to estimate the sex-specific incidence rate of dementia for these risk factors.

**Results:**

502,226 individuals in midlife (54.4% women, mean age 56.5 years) with no prevalent dementia were included in the analyses. Over 11.8 years (median), 4068 participants (45.9% women) developed dementia. The crude incidence rates were 5.88 [95% CI 5.62–6.16] for women and 8.42 [8.07–8.78] for men, per 10,000 person-years. Sex was associated with the risk of dementia, where the risk was lower in women than men (HR = 0.83 [0.77–0.89]). Current smoking, diabetes, high adiposity, prior stroke and low SES were associated with a greater risk of dementia, similarly in women and men. The relationship between blood pressure (BP) and dementia was U-shaped in men but had a dose-response relationship in women: the HR for SBP per 20 mmHg was 1.08 [1.02–1.13] in women and 0.98 [0.93–1.03] in men. This sex difference was not affected by the use of antihypertensive medication at baseline. The sex difference in the effect of raised BP was consistent for dementia subtypes (vascular dementia and Alzheimer’s disease).

**Conclusions:**

Several mid-life cardiovascular risk factors were associated with dementia similarly in women and men, but not raised BP. Future bespoke BP-lowering trials are necessary to understand its role in restricting cognitive decline and to clarify any sex difference.

**Supplementary Information:**

The online version contains supplementary material available at 10.1186/s12916-021-01980-z.

## Background

Dementia is a fast-growing global epidemic—predominately driven by the steep increase of population ageing [1, 2]—posing immense pressure on the public health, social care and fiscal systems [3]. Around 50 million people are living with dementia globally [4], and this is projected to triple by 2050 [2]. The most recent age-standardised global prevalence and death rate related to dementia were both higher in women than men in 2016 [1].

Cardiovascular risk factors are increasingly recognised as contributors to dementia [3], associated with greater risk not only for vascular dementia but also dementia of the Alzheimer’s type [5, 6]. While some cardiovascular risk factors confer differential excess risks on diseases such as myocardial infarction [7] and stroke [8] for women and men, sex differences in the effect of major cardiovascular risk factors for dementia are not well characterised.

Providing that sex is an important modifier for many diseases including Alzheimer’s disease [9], this study sought to examine the sex differences in the association between major cardiovascular risk factors and the risk of all-cause dementia in the UK Biobank. We also assessed whether sex differences varied across age groups and socioeconomic status.

## Methods

The UK Biobank is a large population-based prospective cohort study that recruited 502,489 individuals ages 40–69, between 2006 and 2010 [10]. Individuals aged 40 to 69 were invited to attend one of the 22 centres for baseline assessment, which included questionnaires soliciting lifestyle and medical history, physical and functional measurements. Written informed consent was obtained for all participants electronically. The present analyses excluded participants with prevalent dementia at baseline (*N* = 263). Follow-up for all participants involved linkage with hospital admissions data from England, Scotland, and Wales and the national death register to identify the date of the first known diagnosis of dementia after the date of baseline assessment. Death data and hospital inpatient data were censored on the 30 November 2020, or when death, fatal or non-fatal dementia was recorded, which yielded a median follow-up time of 11.77 years for women and 11.73 years for men.

### Measurement of risk factors

We examined the associations with incident all-cause dementia for a range of major cardiovascular risk factors: blood pressure (systolic blood pressure (SBP), diastolic blood pressure (DBP)), smoking status and intensity, diabetes mellitus (type 1 and type 2), adiposity (body mass index (BMI), waist circumference (WC), waist-to-hip ratio (WHR) and waist-to-height ratio (WHTR)), prior stroke, socioeconomic status (SES) and lipids (total cholesterol, high-density lipoprotein (HDL) cholesterol and low-density lipoprotein (LDL) cholesterol).

Blood pressure was taken at baseline using the Omron HEM-7015IT digital blood pressure monitor by taking the mean of two sitting measures. Based on the American Heart Association (AHA) 2017 guidelines [11], we categorised blood pressure into four groups (normal: SBP < 120 mmHg and DBP < 80 mmHg; elevated: SBP 120–129 mmHg and DBP < 80 mmHg; stage 1 hypertension: SBP 130–139 mmHg or DBP 80–89 mmHg; stage 2 hypertension: SBP ≥ 140 mmHg or DBP ≥ 90 mmHg). Smoking status was self-reported and categorised as never, former, or current smokers. Daily consumption of cigarettes was collected among current smokers. Self-reported diabetes was recorded: if the age at diagnosis was less than 30, and the participant was using insulin, it was classified as having type 1 diabetes, otherwise as type 2 diabetes. BMI was calculated as the weight of the individual in kilogrammes, measured using the Tanita BC-418 MA body composition analyser, divided by the square of the individual’s standing height in metres. BMI was used as a continuous measure, as well as categorised into four groups (underweight: < 18.5 kg/m^2^; healthy weight: 18.5–24.9 kg/m^2^; overweight: 25.0–29.9 kg/m^2^; obese: 30.0 kg/m^2^ and above). Waist and hip circumference were measured using the Wessex non-stretchable sprung tape measure. History of stroke was self-reported, based on a touchscreen questionnaire, and a nurse-led interview was conducted to confirm the medical history. SES was determined using the Townsend Deprivation Index and categorised into three levels using the national cut-off points (high ≤ − 2.08; middle − 2.08 – 1.40; low ≥ 1.40). The Townsend Deprivation Index is a measure of area deprivation, derived from the national census data about unemployment, car ownership, household overcrowding and owner occupation, with higher scores indicate higher levels of deprivation [12]. Blood lipid levels were measured using the Beckman Coulter AU580, and elevated cholesterol was defined as total cholesterol ≥ 6.2 mmol/L.

### Dementia outcome

The primary study endpoint was incident fatal or non-fatal all-cause dementia. The International Classification of Diseases ICD-10 codes (A81.0, F00, F01, F02, F03, F05, G30, G31.0, G31.1, G31.8, and I67.3) were used to identify participants with dementia if one or more of these codes were recorded as a primary or secondary diagnosis in the health records or recorded as the underlying or contributory cause of death in the death registers. Outcome adjudication for incident dementia was conducted by the UK Biobank Outcome Adjudication Group, and ICD-10 codes were used to determine Alzheimer’s disease (AD) (F00, G30) and vascular dementia (F01, I67.3).

### Statistical analysis

Baseline characteristics for women and men were presented as number (percentage) for categorical variables and as mean (standard deviation) for continuous variables. The crude incidence of all-cause dementia was modelled using Poisson regression, with sex as a covariate and a log offset for person-years. Cox proportional hazards regression models were used to estimate the sex-specific hazard ratios (HR) and 95% confidence intervals (CI) for each risk factor for dementia, with interaction terms fitted between each exposure variable and sex [13].

Analyses for all risk factors were adjusted for age, with different sets of covariates, determined a priori, for each risk factor according to perceived probable causal relationships. All models were adjusted for age. In addition, SBP, diabetes, socioeconomic status and total cholesterol were adjusted for each other, and adjusted for smoking status, BMI, lipid-lowering drugs and antihypertensive drugs. For DBP and AHA hypertension, the same adjustments were made as SBP. Stroke and smoking variables were adjusted for socioeconomic status. Body adiposity was adjusted for smoking status and socioeconomic status. HDL and LDL cholesterol were adjusted the same way as total cholesterol. The interaction term between each risk factor and sex was used to obtain the women-to-men ratio of hazard ratios (RHR) for each risk factor [13]. The effects of the risk factors were evaluated separately for vascular dementia and AD using Cox models, with the same adjustments used in the main analyses.

We conducted subgroup analyses investigating whether sex differences in risk factors for dementia varied by age group (< 60 years and ≥ 60 years). Similarly, subgroup analyses were undertaken by SES groups, categorising individuals as above or below the national median Townsend score in the UK (− 0.56).

In addition to making relative comparisons, we evaluated sex differences on the absolute scale, by estimating sex-disaggregated unadjusted and multiple-adjusted rates per 10,000 person-years, and the women-to-men difference of the rate differences, using Poisson regression models. Covariate adjustments were the same as those made in the Cox models.

Post-hoc exploratory analyses were conducted after observing unexpected results from the initial analyses for blood pressure and BMI. We assessed the shape of the associations for continuous SBP, DBP and BMI, with the risk of dementia for women and men using penalised smoothing splines, adjusted for the same set of covariates as outlined for the Cox models. Extreme values in the upper and lower 0.1% of the blood pressure and BMI distributions, which are associated with unreliable estimates, were omitted. The reference was 120.0 mmHg for SBP, 80.0 mmHg for DBP and 22.5 kg/m^2^ for BMI. Sex differences in blood pressure were further explored by disaggregating the results by baseline antihypertensive use.

Kaplan-Meier survival curves were additionally constructed to assess the survival probability of death and dementia by sex.

Sensitivity analysis was conducted, by fitting the proportional sub-distribution hazards regression model described by Fine and Grey [14], in which death was considered as a competing risk.

All analyses were performed on complete case data using R Studio Version 4.0.2 (R Core Team, 2020) and Stata 16.0 (StataCorp, 2019).

## Results

A total of 502,226 individuals (54.4% women) without a prior diagnosis of dementia were included in the analyses. The mean age of participants was 56.3 years (standard deviation (SD) = 8.0) for women and 56.7 years (SD = 8.2) for men (Table 1). On average, women had lower SBP and DBP and a lower percentage of women than men had diabetes and stroke at study baseline.

**Table 1:** Baseline characteristics of women and men in UK Biobank

Characteristics	Women (***n*** = 273,262)	Men (***n*** = 228,964)
Age (years) (mean (SD))	56.3 (8.0)	56.7 (8.2)
Ethnicity:		
White	257,320 (94.2)	215,111 (93.9)
Other	14,678 (5.8)	12,344 (6.1)
Blood pressure (mmHg):		
Systolic (mean (SD))	135.3 (19.2)	140.9 (17.5)
Diastolic (mean (SD))	80.7 (10.0)	84.1 (10.0)
AHA hypertension categories:		
Normal	55,316 (20.2)	20,020 (8.7)
Elevated	35,561 (13.0)	26,008 (11.4)
Stage 1 hypertension	72,038 (26.4)	62,661 (27.4)
Stage 2 hypertension	109,578 (40.1)	119,722 (52.3)
Smoking status:		
Never smoker	161,979 (59.3)	111,404 (48.7)
Former smoker	85,412 (31.3)	87,535 (38.2)
Current smoker	24,359 (8.9)	28,593 (12.5)
By smoking intensity:		
1–9 cigarettes per day	4397 (18.1)	2805 (9.8)
10–19 cigarettes per day	8440 (34.7)	6996 (24.5)
≥ 20 cigarettes per day	5590 (22.9)	7908 (27.7)
Not reported	5932 (24.4)	10,884 (38.1)
Diabetes:		
Type 1 diabetes*	564 (0.2)	652 (0.3)
Type 2 diabetes	9946 (3.6)	15,514 (6.8)
Body mass index (kg/m^2^):		
Body mass index (mean (SD))	27.1 (5.2)	27.8 (4.2)
BMI categories:		
Underweight (< 18.5)	2079 (0.8)	547 (0.2)
Healthy weight (18.5–24.9)	105,618 (38.7)	56,710 (24.8)
Overweight (25.0–29.9)	99,828 (36.5)	112,175 (49.0)
Obese (30.0 and above)	64,282 (23.5)	57,888 (25.3)
Waist circumference (cm) (mean (SD))	84.7 (12.6)	97.0 (11.4)
Waist-to-hip ratio (mean (SD))	0.82 (0.07)	0.94 (0.07)
Waist-to-height ratio (mean (SD))	0.52 (0.08)	0.55 (0.07)
History of stroke	3143 (1.2)	4500 (2.0)
Socioeconomic status (Townsend score thirds):		
High	138,673 (50.7)	115,578 (50.5)
Middle	81,866 (30.0)	66,667 (29.1)
Low	52,396 (19.2)	46,423 (20.3)
Lipids (mmol/L):		
Total cholesterol (mean (SD))	5.9 (1.1)	5.5 (1.1)
HDL (mean (SD))	1.6 (0.4)	1.3 (0.3)
LDL (mean (SD))	3.6 (0.9)	3.5 (0.9)
Elevated cholesterol	93,062 (34.1)	54,507 (23.8)
Drug use:		
Antihypertensive drugs	38,406 (14.1)	47,966 (20.9)
Lipid-lowering drugs	29,503 (10.8)	45,731 (20.0)

*SD* standard deviation, *AHA* American Heart Association, *HDL* high-density lipoprotein, *LDL* low-density lipoprotein

*Defined as diagnosis before the age of 30 and receiving insulin treatment

Over a median 11.8 years follow-up, 4068 (1866 women) cases of incident all-cause dementia were documented.

### Sex comparison of risk factor associations

Sex was a significant risk factor for dementia, with women at a lower risk of dementia than men (HR, 0.83 [0.77–0.89]) after multiple adjustments of systolic blood pressure, baseline age, smoking status, lipids medications, total cholesterol, antihypertensive medications, body mass index, diabetes and Townsend deprivation index.

**Fig. 1:**
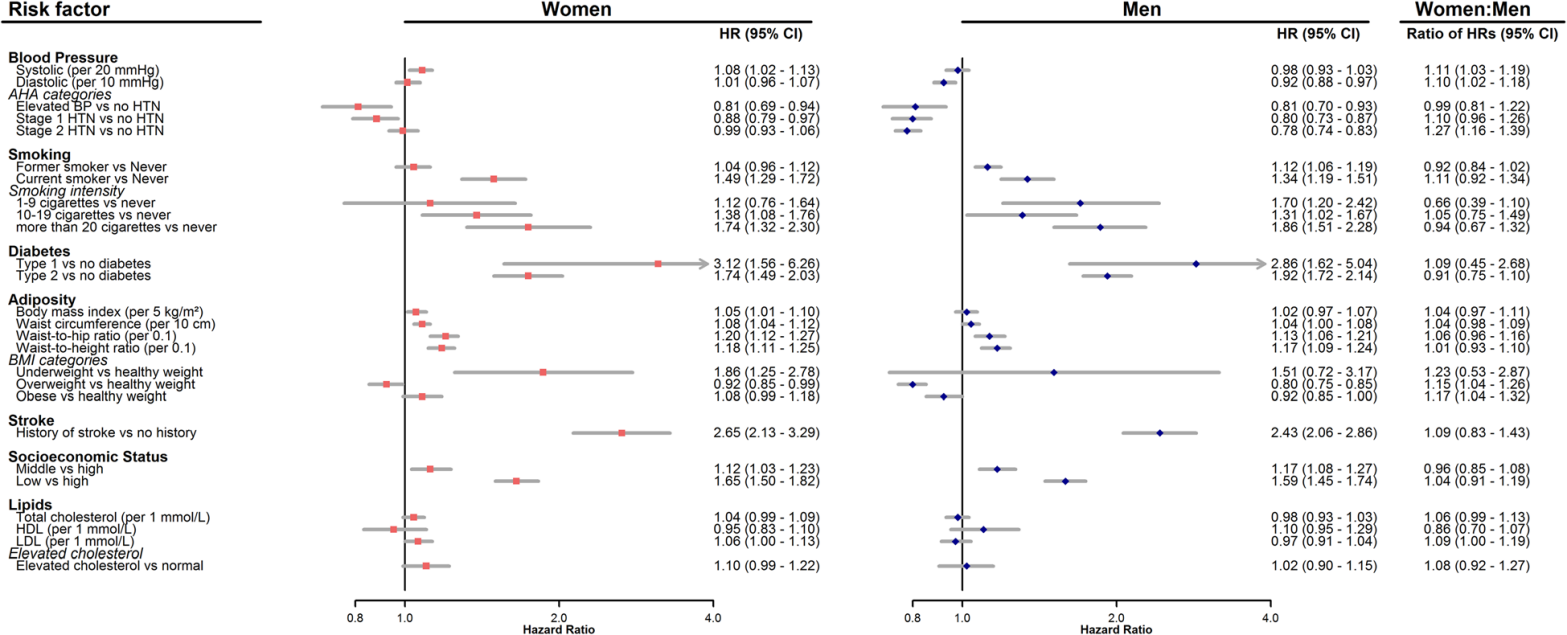
Sex-specific multiple-adjusted hazard ratios and women-to-men ratio of hazard ratios for risk factors and dementia. HR, hazard ratio; CI, confidence interval; AHA, American Heart Association; BP, blood pressure; HTN, hypertension; BMI, body mass index; SES, socioeconomic status; HDL, high-density lipoprotein; LDL, low-density lipoprotein. Pink squares represent hazard ratios for women, and blue diamond represent hazard ratios for men, horizontal lines indicate corresponding 95% confidence intervals around hazard ratios. Hazard ratios for systolic blood pressure is given per 20 mmHg and diastolic blood pressure per 10 mmHg; BMI is given per 5 kg/m^2^, waist circumference is given per 10 cm, waist-to-hip ratio and waist-to-height ratio are given per 0.1 increase in ratio; lipids are given per 1 mmol/L. Hazard ratios were calculated from separate models with different sets of covariate adjustment. All models were adjusted for age. In addition, SBP, diabetes, socioeconomic status and total cholesterol were adjusted for each other, as well as smoking status, body mass index, lipid-lowering drugs and antihypertensive drugs. Same adjustments were made for DBP and AHA hypertension as for SBP. History of stroke and smoking variables were adjusted for socioeconomic status. Body adiposity variables were adjusted for smoking status and socioeconomic status. HDL, LDL cholesterol and elevated cholesterol were adjusted the same way as total cholesterol

The age-adjusted HRs (see Additional file 1) are broadly similar to the multiple adjusted HRs (Fig. 1), although the associations between lipids and dementia were no longer significant after multiple adjustments.

#### Blood pressure

**Fig. 2:**
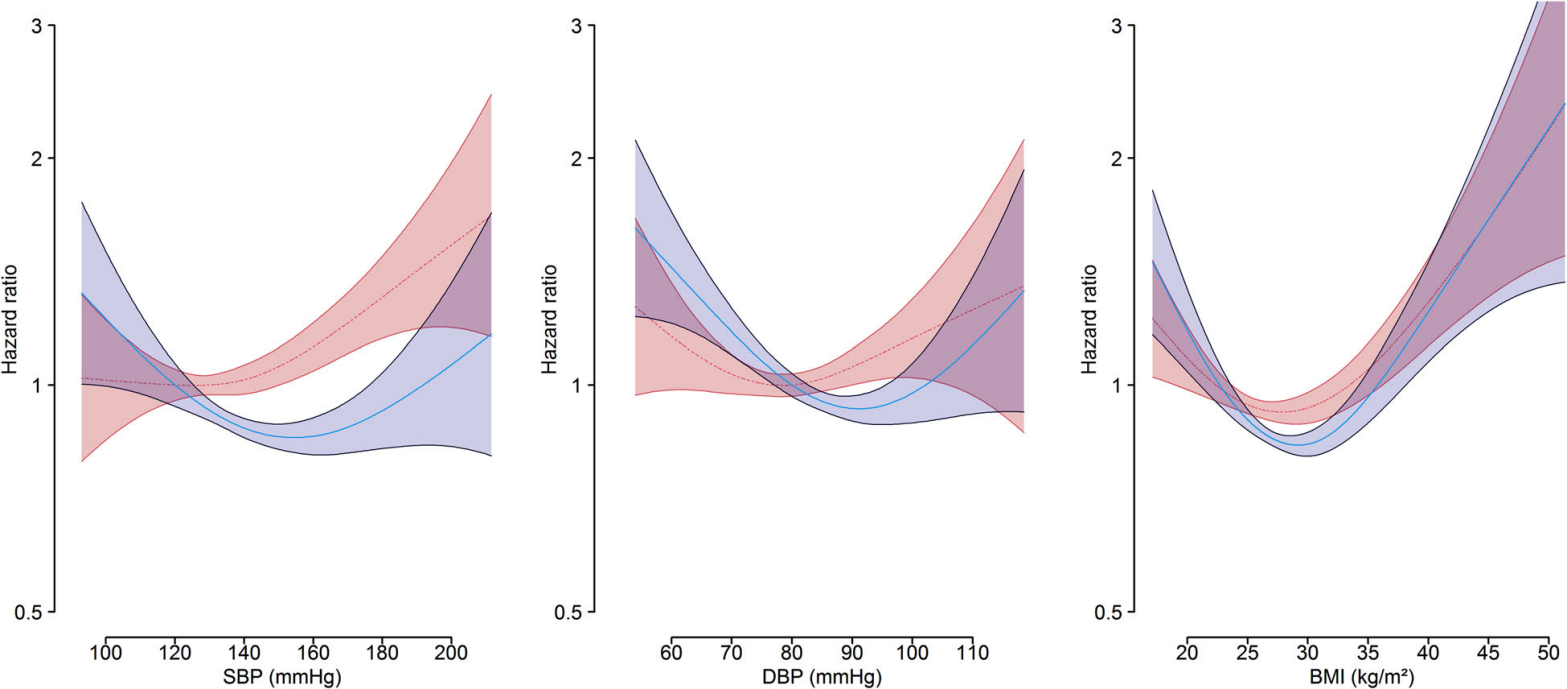
Sex-specific multiple-adjusted hazard ratios for continuous BMI and blood pressure with the risk of dementia. HR, hazard ratio; SBP, systolic blood pressure; DBP, diastolic blood pressure; BMI, body mass index. Modelled with penalised smoothing splines. Splines for systolic and diastolic blood pressure were adjusted for age, smoking status, body mass index, diabetes status, total cholesterol, socioeconomic status, lipid-lowering drugs and antihypertensive drugs. Splines for BMI were adjusted for age, smoking status, and socioeconomic status. Reference value for systolic blood pressure was 120.0 mmHg, and for diastolic blood pressure was 80.0 mmHg. The pink dotted line represents the hazard function for women, and the pink shaded area is the 95% confidence intervals for women; the blue line represents the hazard function for men, and the blue shaded area is the 95% confidence intervals for men. Extreme values in the upper and lower 0.1% of the blood pressure distribution were excluded (systolic blood pressure range: 93.0 mmHg to 211.5 mmHg; diastolic blood pressure range: 54.0 mmHg to 118.5 mmHg). Reference value for BMI was 22.5 kg/m^2^. The pink dotted line represents the hazard function for women, and the pink shaded area is the 95% confidence intervals for women; the blue line represents the hazard function for men, and the blue shaded area is the 95% confidence intervals for men. Extreme values in the upper and lower 0.1% of the BMI distribution were excluded (BMI range 17.1 kg/m^2^ to 51.4 kg/m^2^)

Higher values of SBP were associated with a greater risk of dementia in women (HR, 1.08 [1.02–1.13] per 20 mmHg), but not in men (0.98 [0.93–1.03]); DBP was associated with lower risk of dementia in men (0.92 [0.88–0.97] per 10 mmHg), but not in women (1.01 [0.96–1.07]). Compared with normal blood pressure, men had a lower risk of dementia in different stages of hypertension, whereas the risk increased as hypertension stages worsen in women. The spline analysis suggested that the HR of dementia in women tended to increase with SBP, whereas there was a U-shaped relationship for men. For diastolic blood pressure, there was a clear U-shaped relationship with the risk of dementia for both women and men (Fig. 2). In those without antihypertensive use at baseline (*n* = 415,854), the relationships between blood pressure and the risk of dementia remained similar to the overall association (see Additional file 2).

#### Smoking

Compared with never smoking, the HR for current smoking was 1.49 [1.29–1.72] in women and 1.34 [1.19–1.51] in men; for former smoking, the HR was 1.04 [0.96–1.12] in women and 1.12 [1.06–1.19] in men. There was no evidence of a sex difference in dementia risk by smoking status nor by smoking intensity.

#### Diabetes

For type 1 diabetes, the HR of dementia was 3.12 [1.56–6.26] in women and 2.86 [1.62–5.04] in men. For type 2 diabetes, the HR was 1.74 [1.49–2.03] in women and 1.92 [1.72–2.14] in men. There was no evidence of any sex difference.

#### Adiposity

The HR for dementia associated with BMI per 5 kg/m^2^ was 1.05 [1.01–1.10] in women and 1.02 [0.97–1.07] in men. For WC, the HR per 10 cm was 1.08 [1.04–1.12] in women and 1.04 [1.00–1.08] in men. For WHR, the HR per 0.1 was 1.20 [1.12–1.27] in women and 1.13 [1.06–1.21] in men. For WHTR, the HR per 0.1 was 1.18 [1.11–1.25] in women and 1.17 [1.09–1.24] in men. For BMI categories, the HR for underweight in comparison to healthy weight was 1.86 [1.25–2.78] in women and 1.51 [0.72–3.17] in men, and the HR for overweight was 0.92 [0.85–0.99] in women and 0.80 [0.75–0.85] in men; the HR for obese was 1.08 [0.99–1.18] in women and 0.92 [0.85–1.00] in men. There was evidence for a sex difference among those who were overweight and obese in comparison to a healthy weight, with women in these groups had a greater risk of dementia (women-to-men RHR, 1.15 [1.04–1.26] for overweight, and 1.17 [1.04–1.32] for obese). The splines revealed J-shaped associations between BMI and the risk of dementia for both women and men (Fig. 2).

#### Stroke

The HR for dementia associated with prior stroke was 2.65 [2.13–3.29] in women and 2.43 [2.06–2.86] in men, and there was no evidence for a sex difference.

#### Socioeconomic status

Compared with the highest SES, the HR for the lowest SES was 1.65 [1.50–1.82] in women and 1.59 [1.45–1.74] in men, and the HR for the middle SES was 1.12 [1.03–1.23] in women and 1.17 [1.08–1.27] in men. No sex difference was evident.

#### Lipids

For each of total, HDL and LDL cholesterol, there was no evidence of an association with dementia in women or in men, except for LDL cholesterol in women; the HR was 1.06 [1.00–1.13] per 1 mmol/L for women whilst this was 0.97 [0.91–1.04] for men, and there was some evidence indicated LDL cholesterol was associated with a greater risk of dementia in women in comparison to men (women-to-men RHR, 1.09 [1.00–1.19]).

The C-statistics from multiple-adjusted Cox models indicated good discrimination of these models (see Additional file 3).

### Risk factors by dementia subtypes

The results for vascular dementia and AD were broadly similar to all-cause dementia (see Additional file 4). There was some evidence indicating that diabetes, smoking and stroke may be more strongly associated with vascular dementia than with AD. The sex difference in the effect of raised BP was consistent for vascular dementia and AD.

### Modification by age

There was no evidence for effect modification of the sex differences by age group (≥ 60 vs < 60 years), except for among those who smoked ≥ 20 cigarettes per day; it appeared that the risk of dementia was higher in men among the younger age group, while the risk of dementia was higher in women among the older age group (*p* = 0.03) (see Additional file 5).

### Modification by socioeconomic status

There was some evidence of heterogeneity by SES in sex differences between blood pressure (systolic and diastolic) and dementia (*p* = 0.03 for SBP and *p* = 0.01 for DBP; see Additional file 6), driven by the comparatively stronger association of blood pressure with dementia among women of higher SES.

### Sex comparison of rates of dementia

The crude incidence rates for dementia was 5.88 [5.62–6.16] in women and 8.42 [8.07–8.78] in men per 10,000 person-years. For all risk factors considered, and for all the categories in these risk factors, the rates of dementia were higher in men than women (Table 2). Unadjusted rates were broadly similar to the multiple-adjusted rates (see Additional file 7).

**Table 2:** Sex-specific multiple-adjusted rates of dementia and women-to-men difference of rate differences for each risk factor

Variables	Rates/10,000 person years (95% CI)		Difference of rate differences (95% CI)
	Women (***n*** = 273,262)	Men (***n*** = 228,964)	
AHA hypertension categories:			
Normal	6.43 (5.49, 7.37)	8.71 (7.32, 10.11)	–
Elevated	5.23 (4.43, 6.02)	7.24 (6.20, 8.27)	− 0.27 (− 2.37, 1.82)
Stage 1 hypertension	5.69 (5.10, 6.28)	7.17 (6.49, 7.85)	− 0.80 (− 2.68, 1.08)
Stage 2 hypertension	6.44 (6.00, 6.87)	7.05 (6.63, 7.47)	− 1.67 (− 3.45, 0.11)
Smoking status:			
Never smoker*	5.78 (5.42, 6.13)	7.27 (6.77, 7.76)	–
Former smoker	5.92 (5.46, 6.38)	7.99 (7.50, 8.47)	0.57 (− 0.33, 1.48)
Current smoker	8.11 (6.93, 9.29)	8.98 (7.89, 10.06)	− 0.63 (− 2.35, 1.09)
Smoking intensity (average number of cigarettes smoked daily):			
Never smoker**	5.19 (4.87, 5.51)	6.53 (6.08, 6.97)	–
1–9 cigarettes	5.58 (3.43, 7.72)	10.47 (6.78, 14.17)	3.56 (− 0.75, 7.87)
10–19 cigarettes	6.76 (5.09, 8.43)	7.82 (5.86, 9.77)	− 0.28 (−2.91, 2.36)
20 cigarettes	8.19 (5.91, 10.47)	10.56 (8.37, 12.75)	1.04 (− 2.18, 4.25)
Diabetes:			
No diabetes	5.78 (5.46, 6.10)	6.74 (6.39, 7.08)	–
Type 1^#^	15.74 (4.77, 26.71)	17.63 (7.58, 27.67)	0.93 (− 13.96, 15.81)
Type 2	9.72 (8.12, 11.33)	12.34 (10.80, 13.89)	1.66 (− 0.62, 3.94)
Body mass index:			
Healthy weight (18.5–24.9)	6.03 (5.55, 6.50)	8.68 (7.97, 9.40)	–
Underweight (< 18.5)	10.52 (6.30, 14.73)	10.68 (2.75, 18.60)	− 2.50 (− 11.51, 6.52)
Overweight (25.0–29.9)	5.57 (5.15, 6.00)	7.01 (6.57, 7.46)	− 1.22 (− 2.27, − 0.16)
Obese (30.0 and above)	6.67 (6.09, 7.24)	8.16 (7.50, 8.82)	− 1.16 (− 2.39, 0.07)
Stroke:			
None	5.87 (5.60, 6.14)	7.52 (7.19, 7.85)	–
History of stroke	14.64 (11.51, 17.76)	17.05 (14.36, 19.74)	0.76 (− 3.38, 4.91)
Townsend score thirds:			
High	5.37 (4.97, 5.77)	6.35 (5.93, 6.77)	–
Middle	5.98 (5.43, 6.52)	7.30 (6.68, 7.92)	0.34 (− 0.65, 1.32)
Low	8.69 (7.85, 9.53)	9.60 (8.70, 10.51)	− 0.07 (− 1.42, 1.29)
Lipids:			
Normal cholesterol	6.04 (5.66, 6.42)	7.33 (6.95, 7.70)	–
Elevated cholesterol	6.58 (6.08, 7.08)	7.91 (7.22, 8.59)	0.04 (− 0.98, 1.06)

*AHA* American Heart Association

All models were adjusted for age. Hypertension was adjusted for smoking status, total cholesterol, body mass index, diabetes, socioeconomic status, lipid-lowering drugs and antihypertensive drugs. Diabetes was adjusted for smoking status, body mass index, systolic blood pressure, total cholesterol, socioeconomic status, lipid-lowering drugs and antihypertensive drugs. Socioeconomic status was adjusted for smoking status, systolic blood pressure, total cholesterol, body mass index, diabetes, lipid-lowering drugs and antihypertensive drugs. Body mass index was adjusted for smoking status and socioeconomic status. Elevated cholesterol was adjusted for smoking status, systolic blood pressure, body mass index, socioeconomic status, lipid-lowering drugs and antihypertensive drugs

*Models for smoking status (*) and smoking intensity (**) were fitted using different variables, given the model for smoking intensity excluded former smokers and those with missing cigarette consumption, and thus produced slightly different multiple-adjusted rates of dementia for the never smoker category compared to the rates in the model for smoking status

^#^Defined as diagnosis before the age of 30 and receiving insulin treatment

In addition, the Kaplan-Meier survival curves indicated that the survival probabilities of both death and dementia were lower for men in comparison with women throughout follow-up (see Additional file 8).

### Competing risk of death

The competing risk of death was considered in the analyses, and the results are presented in the supplementary material (see Additional file 9). There were minor differences between the competing risk analysis and the main analysis presented in Fig. 1. Most notably, the ratios of sub-distribution hazard ratios for BMI categories were no longer significant compared with our main analysis.

## Discussion

This study of over half a million participants in the UK Biobank assessed the presence of sex differences in major cardiovascular risk factors and the risk of incident all-cause dementia. On average, men have more adverse cardiovascular profile compared with women at the study baseline. Current smoking, type 1 and type 2 diabetes, high adiposity, stroke and low SES were each associated with increased risk of dementia, similarly in women and men. While the rates of dementia were higher in men than women at all levels considered, the relationship between blood pressure and dementia was U-shaped in men but had a dose-response relationship in women, and this difference appeared to be restricted to those of higher SES.

### Blood pressure

High blood pressure has been consistently linked to dementia and impairment of some cognitive domains [15, 16], but few studies have evaluated potential sex differences, with mixed empirical results [17–21]. A study of included members of the Kaiser Permanente Northern California (KPNC) found that midlife hypertension was associated with a higher risk of dementia in women only [17]; with two other studies also reporting that hypertension was associated with vascular dementia [18] and mild cognitive impairment [19] in women but not in men. Our study not only used guideline-based definitions to categorise hypertension, blood pressures were also included as continuous measures, offering insights to more precise blood pressure targets in improving cognitive health.

Several mechanisms may provide some explanations for the sex differences between blood pressure and dementia observed in our study. First, women may have better cerebral autoregulation than men [22], and reduced cerebral perfusion in men may be associated with a higher incidence of orthostatic hypotension hospitalisation [22], which has been associated with a greater risk of dementia [23]. Second, sex differences in medical treatment are possible. Previous studies reported that treatment adherence was generally lower in women than men, which was in part explained by greater polypharmacy and more side effects in women [24].

### Smoking

Cardiovascular pathology is thought to underlie the increased risk of cognitive impairment and dementia among smokers [3], although smokers also have a higher risk of premature death, precluding them from reaching the age at which dementia might develop; as such, potential uncertainty exists in determining this relationship [3]. Similar to our study, a meta-analysis examined the effects of cigarette smoking on dementia showed that current smokers had a greater risk of all-cause dementia, vascular dementia and AD, but not for former smoker when compared with never smokers [25]. Studies that accounted for the competing risk of death found that the effect of smoking attenuated [26]. Our competing risk analysis indicated that in comparison to never smokers, current smokers and smoking more than 20 cigarettes per day remain to be strong risk factors for all-cause dementia in both women and men. Further clarification is necessary for smoking as a risk factor for dementia.

### Diabetes

A previous meta-analysis found that type 2 diabetes was associated with a 60% increased risk of any dementia in both sexes [27]. Similarly, the present study showed that compared to no diabetes, type 1 and type 2 diabetes were associated with an increased risk of dementia in both women and men. The link between diabetes and dementia is alarming, with the number of people with diabetes projected to increase to 68,000 by 2027 in the UK Biobank [10], as it is increasing globally [28]. While routine screening for cognitive dysfunctions in diabetes is necessary [29], novel treatment options and prevention strategies are crucial for reducing the burden of dementia in this at-risk population.

### Body adiposity

The relationship between body adiposity and dementia is complex and appear to be non-linear depending on dementia subtypes [30]. Our recent meta-analysis, with the inclusion of data from the UK Biobank, showed comparable findings where underweight (BMI < 18.5 kg/m^2^) was strongly associated with the risk of both vascular and non-vascular dementia [30]. Although no sex difference was reported in the meta-analysis, these effects were only significant in women [30]. Underweight is an important subclinical consequence of prodromal dementia, such that the possibility of reverse causality cannot be ruled out.

### Stroke

A previous meta-analysis showed that female sex was associated with a 30% greater risk for post-stroke dementia, although this may have been influenced by women’s older age [31]. While there was no evidence for a sex difference in the effects of stroke in the present study, a history of stroke was associated with more than double the risk of dementia in both sexes. Our results complement the joint call for action by the World Stroke Organisation in prevention for stroke and dementia [32], as it was estimated that more than a third of dementia could be prevented by preventing stroke [32].

### Socioeconomic status

The Three-City Study found that women living in deprived neighbourhoods have a greater risk of dementia, but not men [33]. Another study concluded that lower educational attainment was independently associated with a greater risk of dementia death in women, after considering other comorbidities and behaviours [34]. Given deprivation was associated with the risk of dementia to a similar extent in women and men, reducing socioeconomic inequities may thus have significant effects on dementia prevention, as well as many other conditions on a population level.

### Lipids

The evidence for lipids as risk factors for dementia has been mixed, with a meta-analysis suggesting that high total cholesterol in midlife is associated with a greater risk of developing AD [35]. A recent meta-meta-analysis, which explored the effect of cholesterol fractions concluded that LDL cholesterol increased the risk of AD, while HDL and total cholesterol showed a non-significant association with AD risk [36]. A Mendelian Randomisation study confirmed that high circulating total cholesterol and a reduced level of HDL cholesterol might be associated with an increased risk of AD [37]. Our results do not support such associations.

### Strengths and limitations of the study

To our knowledge, this study is the first to systematically evaluate sex differences in a range of cardiovascular risk factors for dementia in a large general population, using standardised methodology. This study was further strengthened by its prospective cohort design and large sample size. Sex differences were compared on both relative and absolute scales, providing insights to inform public health and clinical practice in risk reduction for dementia. This study also has limitations. The UK Biobank cohort is a relatively healthy and affluent population, predominantly of Caucasian ancestry, which may limit the generalisability of the results. Self-reported smoking, diabetes and history of stroke may be subject to reporting bias. Multiple testing of interactions can raise the concern of false positives. With the threshold of 5%, one significant test would be expected in every 20 tests performed, even if there were no real effects. Thus, our results should be interpreted with caution. Lastly, while we were able to distinguish the effects of blood pressure by baseline antihypertensive use, we had no further information on medication use duration or dose, hence limiting our ability to interpret the results.

## Conclusions

Several cardiovascular risk factors were found to be associated with incident dementia in both sexes. Despite the rate of incident dementia being higher in men compared to women, raised blood pressure was associated with a greater relative risk of dementia in women than equivalent men. Given the lack of proven pharmaceutical treatments for dementia, public health strategies to promote healthy lifestyles are important to reduce the burden of dementia. Among trials aim to prevent cognitive decline or dementia, the strongest evidence lies in treating hypertension [3]. However, findings from this research suggests this is not sufficient and warrants further work to identify factors associated with relative poverty that is also important. Assuming causality, bespoke randomised control trials of blood pressure lowering are necessary, to understand its role in attenuating cognitive decline, and such trials should include an equal number of women and men in order to clarify potential sex differences.

## Supplementary Information

**Additional file 1.** Age-adjusted hazard ratios and ratio of the hazard ratios (women-to-men) for risk factors and dementia, by sex.

**Additional file 2.** Multiple adjusted hazard ratios for systolic and diastolic blood pressure and dementia disaggregated by antihypertensive use, by sex.

**Additional file 3.** Multiple-adjusted C-statistics with standard errors (se) for each risk factor in association with all-cause dementia.

**Additional file 4.** Multiple-adjusted hazard ratios and ratio of the hazard ratios (women-to-men) for risk factors and by dementia subtypes.

**Additional file 5.** Multiple-adjusted hazard ratios and women-to-men ratio of hazard ratios for risk factors and dementia, by age group and sex.

**Additional file 6.** Multiple adjusted hazard ratios (HRs) and 95% confidence intervals (CI) for the association between risk factors and incident dementia, by socioeconomic status (SES) and sex.

**Additional file 7.** Unadjusted rates of incident dementia (per 10,000 person years) by sex, and women-to-men difference of rate differences for each risk factor.

**Additional file 8.** Kaplan-Meier survival curves for death and dementia, by sex.

**Additional file 9.** Multiple-adjusted sub-distribution hazard ratios (SHR) and ratio of the sub-distribution hazard ratios (women-to-men) for risk factors for dementia from competing risks analysis.

## Data Availability

The data used in this current study are available from the UK Biobank data resources. Permissions are required in order to gain access to the UK Biobank data resources, subject to successful registration and application process. Further information can be found on the UK Biobank website (https://www.ukbiobank.ac.uk/).

## Abbreviations

AD: Alzheimer’s disease
AHA: American Heart Association
BMI: Body mass index
BP: Blood pressure
CI: Confidence interval
DBP: Diastolic blood pressure
HDL: High-density lipoproteins
HR: Hazard ratio
ICD: International Classification of Disease
LDL: Low-density lipoproteins
RHR: Ratio of hazard ratios
SBP: Systolic blood pressure
SD: Standard deviation
SES: Socioeconomic status
WC: Waist circumference
WHR: Waist to hip ratio
WHTR: Waist to height ratio
