# Supplementary material for: Sex differences in the association between major cardiovascular risk factors in midlife and dementia: a cohort study using data from the UK Biobank
Source: BMC Med. 2021 May 19;19:110. doi: 10.1186/s12916-021-01980-z (PMC8132382; doi:10.1186/s12916-021-01980-z)
Supplement: Supplementary file 1 — Additional file 1. Age-adjusted hazard ratios and ratio of the hazard ratios (women-to-men) for risk factors and dementia, by sex. [file 12916_2021_1980_MOESM1_ESM.docx]

**Additional file 1: Age-adjusted hazard ratios and ratio of the hazard ratios (women-to-men) for risk factors and dementia, by sex.**


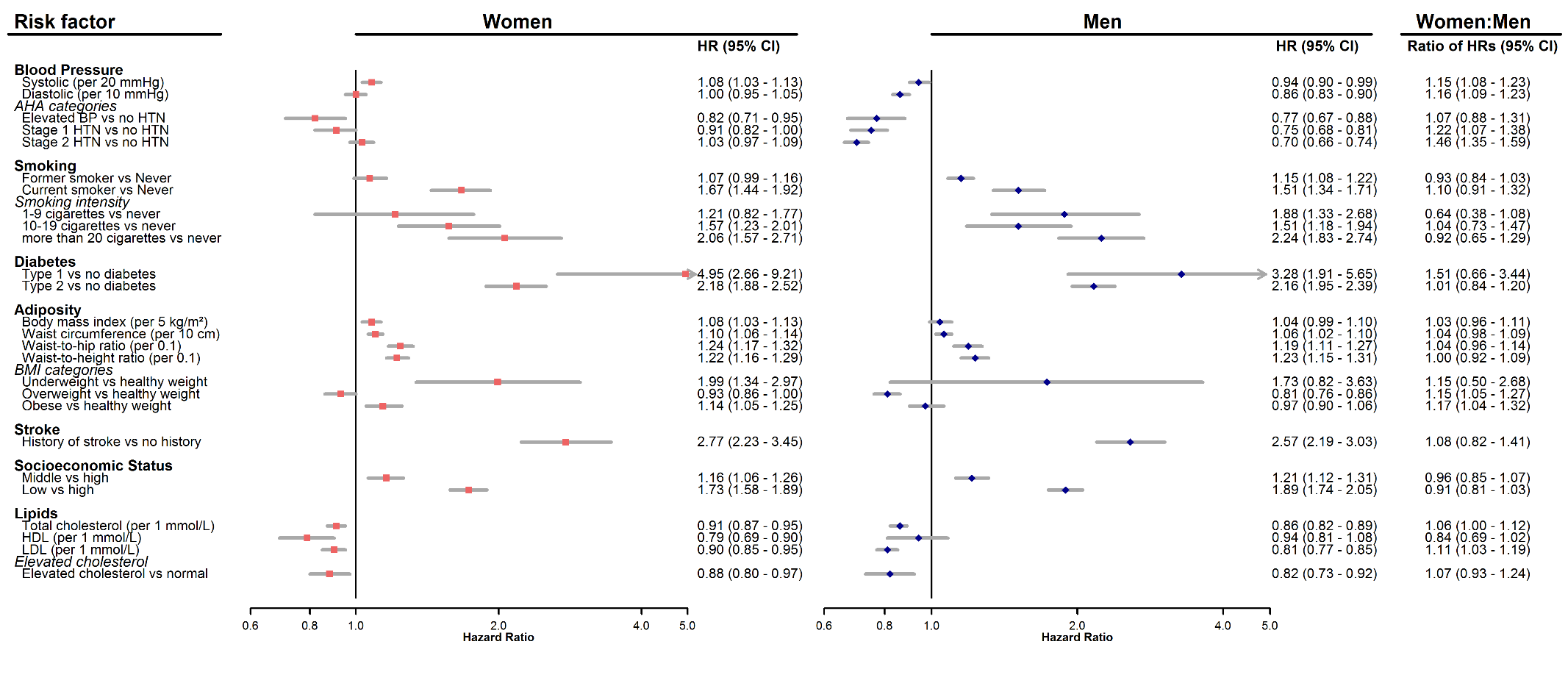


HR, hazard ratio; CI, confidence interval; RHR, ratio of hazard ratios; AHA, American Heart Association; HTN, hypertension; BMI, body mass index; HDL, High-density lipoprotein; LDL, Low-density lipoprotein.

Pink squares represent hazard ratios for women, and blue diamond represent hazard ratios for men, horizontal lines indicate corresponding 95% confidence intervals around hazard ratios. Hazard ratios for systolic blood pressure is given per 20 mmHg and diastolic blood pressure per 10 mmHg; BMI is given per 5 kg/m^2^, waist circumference is given per 10 cm, waist-to-hip ratio and waist-to-height ratio are given per 0.1 increase in ratio; lipids are given per 1 mmol/L.
