# Supplementary material for: Sex differences in the association between major cardiovascular risk factors in midlife and dementia: a cohort study using data from the UK Biobank
Source: BMC Med. 2021 May 19;19:110. doi: 10.1186/s12916-021-01980-z (PMC8132382; doi:10.1186/s12916-021-01980-z)
Supplement: Supplementary file 2 — Additional file 2. Multiple adjusted hazard ratios for systolic and diastolic blood pressure and dementia disaggregated by antihypertensive use, by sex. [file 12916_2021_1980_MOESM2_ESM.docx]

**Additional file 2: Multiple adjusted hazard ratios for systolic and diastolic blood pressure and dementia disaggregated by antihypertensive use, by sex.**


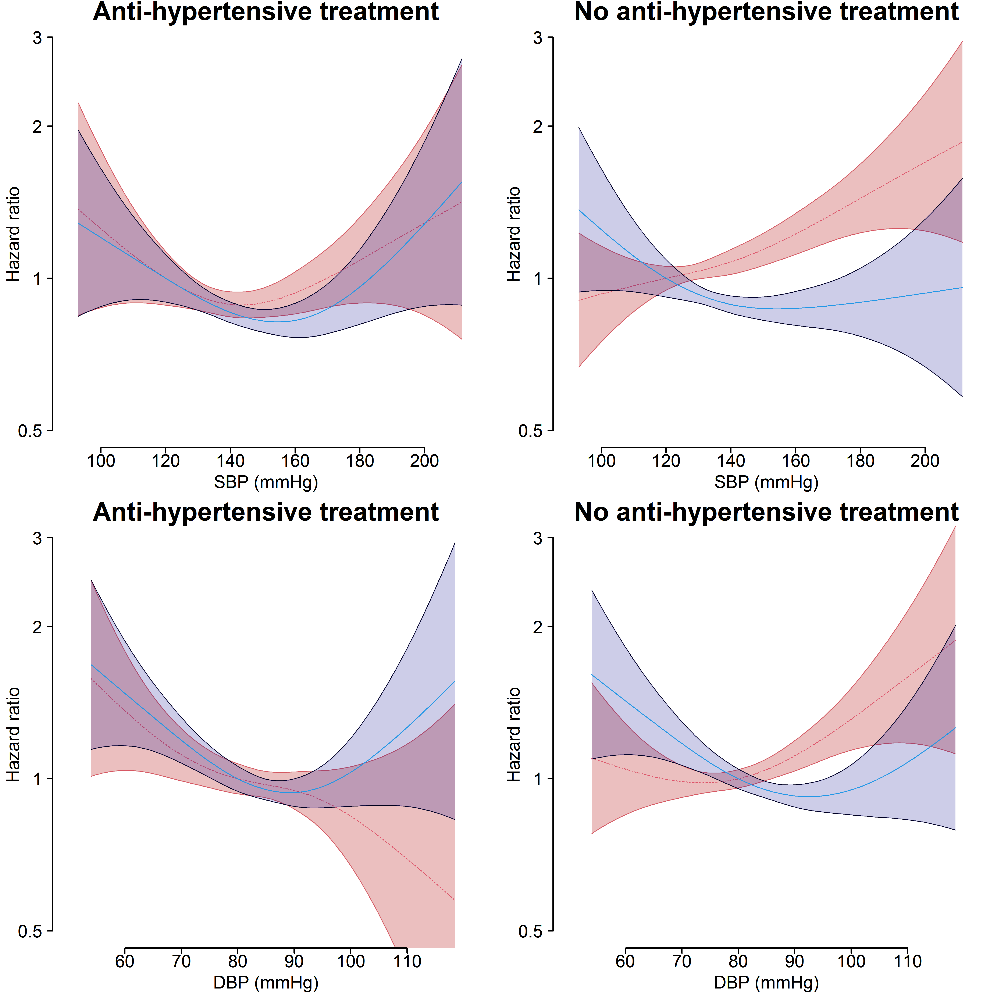


HR, hazard ratio; SBP, systolic blood pressure; DBP, diastolic blood pressure.

Modelled with penalised smoothing splines. Splines for systolic and diastolic blood pressure were adjusted for age, smoking status, body mass index, diabetes status, total cholesterol, socioeconomic status, lipid lowering drugs and antihypertensive drugs.

Reference values for systolic blood pressure was 120.0 mmHg, and for diastolic blood pressure was 80.0 mmHg. The pink dotted line represents the hazard function for women, and the pink shaded area is the 95% confidence intervals for women; the blue line represents the hazard function for men, and the blue shaded area is the 95% confidence intervals for men. Extreme values in the upper and lower 0.1% of the blood pressure distribution were excluded (systolic blood pressure range: 93.0mmHg to 211.5mmHg; diastolic blood pressure range: 54.0mmHg to 118.5mmHg).
