# Supplementary material for: Sex differences in the association between major cardiovascular risk factors in midlife and dementia: a cohort study using data from the UK Biobank
Source: BMC Med. 2021 May 19;19:110. doi: 10.1186/s12916-021-01980-z (PMC8132382; doi:10.1186/s12916-021-01980-z)
Supplement: Supplementary file 3 — Additional file 3. Multiple-adjusted C-statistics with standard errors (se) for each risk factor in association with all-cause dementia. [file 12916_2021_1980_MOESM3_ESM.docx]

**Additional file 3: Multiple-adjusted C-statistics with standard errors (se) for each risk factor in association with all-cause dementia.**

| **Risk factors** | **C-statistics (se)** |
| --- | --- |
| Systolic blood pressure | 0.805 (0.003) |
| Diastolic blood pressure | 0.805 (0.003) |
| AHA categories of hypertension | 0.805 (0.003) |
| Smoking status | 0.795 (0.003) |
| Smoking intensity | 0.797 (0.004) |
| Diabetes | 0.805 (0.003) |
| Body mass index | 0.796 (0.003) |
| Waist circumference | 0.796 (0.003) |
| Waist to hip ratio | 0.796 (0.003) |
| Waist to height ratio | 0.796 (0.003) |
| Body mass index category | 0.797 (0.003) |
| History of stroke | 0.797 (0.003) |
| Townsend thirds | 0.805 (0.003) |
| Total cholesterol | 0.805 (0.003) |
| HDL cholesterol | 0.805 (0.003) |
| LDL cholesterol | 0.805 (0.003) |
| Elevated cholesterol | 0.805 (0.003) |

AHA, American Heart Association; HDL, high density lipoprotein; LDL, low density lipoprotein.
