# Supplementary material for: Sex differences in the association between major cardiovascular risk factors in midlife and dementia: a cohort study using data from the UK Biobank
Source: BMC Med. 2021 May 19;19:110. doi: 10.1186/s12916-021-01980-z (PMC8132382; doi:10.1186/s12916-021-01980-z)
Supplement: Supplementary file 4 — Additional file 4. Multiple-adjusted hazard ratios and ratio of the hazard ratios (women-to-men) for risk factors and by dementia subtypes. [file 12916_2021_1980_MOESM4_ESM.docx]

**Additional file 4: Multiple-adjusted hazard ratios and ratio of the hazard ratios (women-to-men) for risk factors and by dementia subtypes.**

|  | **Vascular dementia** | | | **Alzheimer’s disease** | | |
| --- | --- | --- | --- | --- | --- | --- |
| **Risk factors** | **Women**  **HR (95% CI)** | **Men**  **HR (95% CI)** | **Women-to-men RHR (95% CI)** | **Women**  **HR (95% CI)** | **Men**  **HR (95% CI)** | **Women-to-men**  **RHR (95% CI)** |
| Systolic blood pressure (per 20mmHg) | 1.11 (1.00, 1.23) | 1.01 (0.91, 1.11) | 1.10 (0.95, 1.27) | 1.10 (1.01, 1.20) | 1.02 (0.93, 1.12) | 1.08 (0.96, 1.22) |
| Diastolic blood pressure (per 10mmHg) | 1.02 (0.92, 1.14) | 0.91 (0.83, 0.99) | 1.13 (0.98, 1.29) | 1.01 (0.93, 1.10) | 0.91 (0.84, 0.99) | 1.11 (0.98, 1.25) |
| AHA categories: |  |  |  |  |  |  |
| Elevated blood pressure vs Normal blood pressure | 0.88 (0.63, 1.24) | 0.95 (0.73, 1.23) | 0.93 (0.61, 1.43) | 1.16 (0.91, 1.49) | 1.00 (0.78, 1.29) | 1.16 (0.81, 1.65) |
| Stage 1 hypertension vs Normal blood pressure | 1.17 (0.95, 1.44) | 0.85 (0.71, 1.02) | 1.37 (1.04, 1.80) | 1.29 (1.09, 1.52) | 1.10 (0.94, 1.29) | 1.17 (0.93, 1.47) |
| Stage 2 hypertension vs Normal blood pressure | 1.25 (1.09, 1.43) | 0.85 (0.76, 0.96) | 1.46 (1.22, 1.74) | 1.36 (1.22, 1.52) | 0.99 (0.89, 1.10) | 1.38 (1.18, 1.61) |
| Former smoker vs Never smoker | 1.07 (0.92, 1.26) | 1.25 (1.12, 1.40) | 0.86 (0.71, 1.04) | 1.05 (0.93, 1.19) | 1.02 (0.92, 1.14) | 1.02 (0.87, 1.21) |
| Current smoker vs Never smoker | 1.39 (1.00, 1.94) | 1.44 (1.14, 1.82) | 0.97 (0.64, 1.45) | 1.10 (0.82, 1.47) | 1.12 (0.88, 1.42) | 0.98 (0.67, 1.42) |
| Smoking intensity: |  |  |  |  |  |  |
| 1-9 cigarettes per day vs Never smoker | 1.00 (0.42, 2.40) | 0.68 (0.22, 2.12) | 1.46 (0.35, 6.10) | 1.00 (0.50, 2.00) | 1.40 (0.70, 2.79) | 0.72 (0.27, 1.91) |
| 10-19 cigarettes per day vs Never smoker | 0.95 (0.49, 1.82) | 1.33 (0.80, 2.20) | 0.71 (0.31, 1.62) | 0.91 (0.54, 1.53) | 0.73 (0.41, 1.32) | 1.24 (0.56, 2.73) |
| ≥ 20 cigarettes per day vs Never smoker | 1.40 (0.70, 2.80) | 2.61 (1.82, 3.75) | 0.54 (0.24, 1.17) | 0.95 (0.50, 1.84) | 1.10 (0.68, 1.77) | 0.87 (0.39, 1.96) |
| Diabetes* vs No diabetes | 1.95 (1.37, 2.79) | 2.34 (1.86, 2.94) | 0.84 (0.55, 1.28) | 1.78 (1.30, 2.44) | 1.60 (1.24, 2.06) | 1.11 (0.74, 1.67) |
| Body mass index (per 5kg/m^2^) | 1.01 (0.92, 1.12) | 1.02 (0.92, 1.13) | 0.99 (0.86, 1.14) | 0.92 (0.85, 1.00) | 0.79 (0.71, 0.87) | 1.17 (1.02, 1.33) |
| Waist circumference (per 10 cm) | 1.01 (0.94, 1.10) | 1.05 (0.97, 1.13) | 0.97 (0.87, 1.08) | 0.97 (0.91, 1.03) | 0.86 (0.80, 0.93) | 1.13 (1.02, 1.24) |
| Waist to hip ratio (per 0.1) | 1.11 (0.97, 1.28) | 1.23 (1.08, 1.39) | 0.91 (0.75, 1.09) | 1.07 (0.95, 1.19) | 0.85 (0.76, 0.96) | 1.25 (1.06, 1.47) |
| Waist to height ratio (per 0.1) | 1.09 (0.96, 1.24) | 1.22 (1.08, 1.38) | 0.90 (0.75, 1.07) | 0.98 (0.88, 1.09) | 0.84 (0.74, 0.95) | 1.17 (0.99, 1.37) |
| BMI categories: |  |  |  |  |  |  |
| Underweight vs Healthy weight | 1.21 (0.45, 3.23) | 2.50 (0.80, 7.77) | 0.48 (0.11, 2.17) | 1.22 (0.55, 2.71) | 2.05 (0.66, 6.35) | 0.60 (0.15, 2.38) |
| Overweight vs Healthy weight | 0.73 (0.62, 0.85) | 0.77 (0.68, 0.87) | 0.94 (0.77, 1.16) | 0.83 (0.73, 0.94) | 0.76 (0.68, 0.85) | 1.09 (0.93, 1.29) |
| Obese vs Healthy weight | 0.90 (0.74, 1.08) | 1.00 (0.86, 1.17) | 0.90 (0.70, 1.14) | 0.78 (0.66, 0.92) | 0.66 (0.56, 0.79) | 1.17 (0.92, 1.48) |
| History of stroke vs No history | 3.24 (2.16, 4.86) | 4.06 (3.15, 5.22) | 0.80 (0.50, 1.29) | 2.10 (1.41, 3.13) | 1.50 (1.04, 2.15) | 1.40 (0.82, 2.41) |
| Middle SES vs High SES | 0.87 (0.71, 1.07) | 1.25 (1.07, 1.46) | 0.70 (0.54, 0.90) | 0.99 (0.85, 1.16) | 1.13 (0.98, 1.31) | 0.88 (0.71, 1.09) |
| Low SES vs High SES | 1.43 (1.16, 1.77) | 1.56 (1.30, 1.86) | 0.92 (0.70, 1.21) | 1.51 (1.27, 1.79) | 1.47 (1.24, 1.74) | 1.03 (0.81, 1.31) |
| Total cholesterol (per 1 mmol/L) | 1.08 (0.98, 1.20) | 1.05 (0.95, 1.15) | 1.04 (0.90, 1.19) | 1.08 (1.00, 1.17) | 0.99 (0.91, 1.08) | 1.09 (0.97, 1.22) |
| HDL cholesterol (per 1 mmol/L) | 1.07 (0.79, 1.44) | 1.09 (0.81, 1.46) | 0.98 (0.64, 1.49) | 1.05 (0.82, 1.33) | 0.81 (0.61, 1.07) | 1.30 (0.89, 1.88) |
| LDL cholesterol (per 1 mmol/L) | 1.11 (0.98, 1.26) | 1.07 (0.95, 1.21) | 1.04 (0.87, 1.24) | 1.11 (1.00, 1.23) | 1.01 (0.91, 1.14) | 1.09 (0.94, 1.27) |
| Elevated cholesterol vs Normal cholesterol | 1.21 (0.96, 1.52) | 1.40 (1.11, 1.78) | 0.86 (0.62, 1.20) | 1.18 (0.99, 1.41) | 1.08 (0.87, 1.33) | 1.09 (0.83, 1.44) |

AHA, American Heart Association; BMI, body mass index; SES, socioeconomic status.

* Diabetes included both type 1 and type 2 diabetes due to small number of events.

Systolic blood pressure, diastolic blood pressure, body mass index, waist circumference, waist-to-hip ratio, waist-to-height ratio and lipids were given per increments (20 mmHg for systolic blood pressure, 10 mmHg for diastolic blood pressure, 5kg/m^2^ for BMI, 10 cm for waist circumference, 0.1 for waist-to-hip ratio, 0.1 for waist-to-height ratio, 1 mmol/L for lipids).

Hypertension stages were compared to participants with normal blood pressure; current and former smokers were compared with never smokers; participants with diabetes were compared with those without diabetes; underweight, overweight and obese were compared with healthy weight (BMI 18.5 – 24.9 kg/m^2^); participants with stroke were compared to those without stroke; and for socioeconomic status the lowest third and middle third were compared with the highest third; elevated cholesterol (total cholesterol ≥ 6.2 mmol/l) was compared to normal cholesterol.

Hazard ratios were calculated from separate models with different sets of covariate adjustment. All models were adjusted for age and Townsend index. In addition, SBP, diabetes and total cholesterol were adjusted for each other, as well as smoking status, body mass index, lipid lowering drugs and antihypertensive drugs. Same adjustments were made for DBP and AHA hypertension as for SBP. History of stroke and smoking variables were adjusted for socioeconomic status. Body adiposity variables were adjusted for smoking. HDL, LDL cholesterol and elevated cholesterol were adjusted the same way as total cholesterol.
