# Supplementary material for: Sex differences in the association between major cardiovascular risk factors in midlife and dementia: a cohort study using data from the UK Biobank
Source: BMC Med. 2021 May 19;19:110. doi: 10.1186/s12916-021-01980-z (PMC8132382; doi:10.1186/s12916-021-01980-z)
Supplement: Supplementary file 5 — Additional file 5. Multiple-adjusted hazard ratios and women-to-men ratio of hazard ratios for risk factors and dementia, by age group and sex. [file 12916_2021_1980_MOESM5_ESM.docx]

**Additional file 5: Multiple-adjusted hazard ratios and women-to-men ratio of hazard ratios for risk factors and dementia, by age group and sex.**

| **Risk factors** | **Age subgroup** | **Women HR (95% CI)** | **Men HR (95% CI)** | **RHR (95% CI)** | **P for interaction** |
| --- | --- | --- | --- | --- | --- |
| Systolic blood pressure (per 20mmHg) | < 60 years | 0.97 (0.84, 1.11) | 1.03 (0.90, 1.19) | 0.94 (0.77, 1.15) | 0.09 |
|  | ≥ 60 years | 1.09 (1.04, 1.16) | 0.97 (0.92, 1.02) | 1.13 (1.05, 1.22) |  |
| Diastolic blood pressure (per 10mmHg) | < 60 years | 0.98 (0.86, 1.11) | 1.01 (0.90, 1.15) | 0.97 (0.81, 1.15) | 0.30 |
|  | ≥ 60 years | 1.02 (0.97, 1.08) | 0.91 (0.86, 0.96) | 1.12 (1.04, 1.21) |  |
| Elevated blood pressure vs Normal blood pressure | < 60 years | 0.83 (0.58, 1.17) | 0.96 (0.68, 1.35) | 0.86 (0.53, 1.40) | 0.61 |
|  | ≥ 60 years | 0.81 (0.68, 0.95) | 0.78 (0.67, 0.91) | 1.03 (0.82, 1.30) |  |
| Stage 1 hypertension vs Normal blood pressure | < 60 years | 0.92 (0.74, 1.14) | 0.80 (0.63, 1.01) | 1.16 (0.84, 1.59) | 0.77 |
|  | ≥ 60 years | 0.87 (0.77, 0.97) | 0.79 (0.72, 0.88) | 1.09 (0.94, 1.27) |  |
| Stage 2 hypertension vs Normal blood pressure | < 60 years | 0.88 (0.71, 1.08) | 0.98 (0.82, 1.16) | 0.90 (0.69, 1.17) | 0.22 |
|  | ≥ 60 years | 1.01 (0.94, 1.09) | 0.75 (0.70, 0.80) | 1.35 (1.23, 1.49) |  |
| Former smoker vs Never smoker | < 60 years | 0.76 (0.60, 0.95) | 1.07 (0.89, 1.30) | 0.70 (0.52, 0.95) | 0.13 |
|  | ≥ 60 years | 1.10 (1.02, 1.19) | 1.13 (1.06, 1.20) | 0.97 (0.88, 1.08) |  |
| Current smoker vs Never smoker | < 60 years | 1.49 (1.12, 1.98) | 1.84 (1.46, 2.33) | 0.81 (0.56, 1.17) | 0.14 |
|  | ≥ 60 years | 1.47 (1.25, 1.74) | 1.22 (1.06, 1.41) | 1.21 (0.97, 1.50) |  |
| 1-9 cigarettes per day vs Never smoker | < 60 years | 1.50 (0.75, 2.99) | 2.14 (1.07, 4.28) | 0.70 (0.26, 1.86) | 0.85 |
|  | ≥ 60 years | 1.01 (0.64, 1.60) | 1.60 (1.06, 2.41) | 0.63 (0.34, 1.17) |  |
| 10-19 cigarettes per day vs Never smoker | < 60 years | 1.63 (1.04, 2.56) | 2.17 (1.43, 3.29) | 0.75 (0.41, 1.39) | 0.27 |
|  | ≥ 60 years | 1.30 (0.97, 1.75) | 1.09 (0.81, 1.48) | 1.19 (0.78, 1.82) |  |
| ≥ 20 cigarettes per day vs Never smoker | < 60 years | 1.19 (0.64, 2.22) | 2.60 (1.84, 3.68) | 0.46 (0.23, 0.94) | 0.03 |
|  | ≥ 60 years | 1.98 (1.45, 2.69) | 1.66 (1.29, 2.14) | 1.19 (0.80, 1.77) |  |
| Type 1 diabetes vs No diabetes | < 60 years | 2.32 (0.58, 9.30) | 4.58 (1.71, 12.25) | 0.51 (0.09, 2.78) | 0.48 |
|  | ≥ 60 years | 3.37 (1.51, 7.51) | 2.40 (1.20, 4.80) | 1.41 (0.49, 4.06) |  |
| Type 2 diabetes vs No diabetes | < 60 years | 1.53 (0.98, 2.38) | 1.80 (1.26, 2.56) | 0.85 (0.48, 1.50) | 0.47 |
|  | ≥ 60 years | 1.76 (1.49, 2.08) | 1.93 (1.72, 2.16) | 0.91 (0.75, 1.12) |  |
| Body mass index (per 5kg/m^2^) | < 60 years | 1.12 (1.02, 1.24) | 1.10 (0.97, 1.24) | 1.03 (0.88, 1.20) | 0.93 |
|  | ≥ 60 years | 1.04 (0.99, 1.09) | 1.01 (0.95, 1.07) | 1.03 (0.96, 1.12) |  |
| Waist circumference (per 10 cm) | < 60 years | 1.16 (1.07, 1.26) | 1.13 (1.03, 1.24) | 1.03 (0.91, 1.16) | 0.99 |
|  | ≥ 60 years | 1.06 (1.02, 1.10) | 1.02 (0.98, 1.07) | 1.03 (0.98, 1.10) |  |
| Waist to hip ratio (per 0.1) | < 60 years | 1.31 (1.19, 1.44) | 1.42 (1.23, 1.65) | 0.92 (0.77, 1.10) | 0.14 |
|  | ≥ 60 years | 1.16 (1.08, 1.24) | 1.09 (1.01, 1.17) | 1.06 (0.96, 1.18) |  |
| Waist to height ratio (per 0.1) | < 60 years | 1.34 (1.18, 1.53) | 1.36 (1.17, 1.59) | 0.99 (0.81, 1.21) | 0.92 |
|  | ≥ 60 years | 1.14 (1.07, 1.22) | 1.13 (1.05, 1.22) | 1.01 (0.92, 1.11) |  |
| Underweight vs Healthy weight | < 60 years | 1.66 (0.62, 4.42) | 3.48 (1.30, 9.35) | 0.48 (0.12, 1.91) | 0.08 |
|  | ≥ 60 years | 1.90 (1.23, 2.95) | 0.85 (0.27, 2.62) | 2.25 (0.67, 7.57) |  |
| Overweight vs Healthy weight | < 60 years | 0.96 (0.78, 1.17) | 0.87 (0.73, 1.03) | 1.10 (0.85, 1.44) | 0.86 |
|  | ≥ 60 years | 0.91 (0.84, 0.99) | 0.79 (0.74, 0.85) | 1.15 (1.03, 1.27) |  |
| Obese vs Healthy weight | < 60 years | 1.29 (1.05, 1.59) | 1.08 (0.88, 1.33) | 1.20 (0.89, 1.60) | 0.35 |
|  | ≥ 60 years | 1.04 (0.94, 1.14) | 0.90 (0.82, 0.99) | 1.15 (1.01, 1.32) |  |
| History of stroke vs No history | < 60 years | 4.87 (2.84, 8.36) | 3.65 (2.26, 5.90) | 1.33 (0.65, 2.75) | 0.55 |
|  | ≥ 60 years | 2.41 (1.90, 3.06) | 2.32 (1.95, 2.76) | 1.04 (0.77, 1.40) |  |
| Middle SES vs High SES | < 60 years | 1.04 (0.82, 1.31) | 1.13 (0.90, 1.41) | 0.92 (0.67, 1.27) | 0.58 |
|  | ≥ 60 years | 1.13 (1.03, 1.25) | 1.18 (1.08, 1.29) | 0.96 (0.85, 1.10) |  |
| Low SES vs High SES | < 60 years | 2.00 (1.61, 2.47) | 1.76 (1.42, 2.19) | 1.13 (0.84, 1.54) | 0.84 |
|  | ≥ 60 years | 1.56 (1.40, 1.74) | 1.55 (1.40, 1.71) | 1.01 (0.87, 1.17) |  |
| Total cholesterol (per 1 mmol/L) | < 60 years | 1.12 (1.00, 1.26) | 1.00 (0.88, 1.12) | 1.13 (0.96, 1.33) | 0.93 |
|  | ≥ 60 years | 1.03 (0.97, 1.08) | 0.98 (0.93, 1.03) | 1.05 (0.98, 1.13) |  |
| HDL cholesterol (per 1 mmol/L) | < 60 years | 0.89 (0.62, 1.28) | 0.85 (0.55, 1.32) | 1.04 (0.59, 1.84) | 0.97 |
|  | ≥ 60 years | 0.98 (0.83, 1.14) | 1.15 (0.98, 1.36) | 0.85 (0.67, 1.06) |  |
| LDL cholesterol (per 1 mmol/L) | < 60 years | 1.15 (1.00, 1.34) | 0.99 (0.84, 1.15) | 1.17 (0.94, 1.45) | 0.96 |
|  | ≥ 60 years | 1.05 (0.98, 1.12) | 0.97 (0.91, 1.04) | 1.08 (0.98, 1.19) |  |
| Elevated cholesterol vs Normal cholesterol | < 60 years | 1.22 (0.94, 1.58) | 1.12 (0.85, 1.47) | 1.09 (0.74, 1.59) | 0.57 |
|  | ≥ 60 years | 1.08 (0.96, 1.22) | 1.00 (0.87, 1.14) | 1.08 (0.91, 1.30) |  |

HR, hazard ratio; CI, confidence interval; RHR, ratio of hazard ratios; SES, socioeconomic status; HDL, High-density lipoprotein; LDL, Low-density lipoprotein.

N = 284 925 in the < 60 years age group, and N = 217 301 in the ≥ 60 years age group.

Hazard ratios were calculated from separate models with different sets of covariate adjustment. All models adjusted for age. SBP, diabetes, socioeconomic status and total cholesterol were adjusted for each other, as well as smoking status, body mass index, lipid lowering drugs and antihypertensive drugs. Same adjustments were made for DBP and AHA hypertension as for SBP. History of stroke and smoking variables were adjusted for socioeconomic status. Body adiposity variables were adjusted for smoking status and socioeconomic status. HDL, LDL cholesterol and elevated cholesterol were adjusted the same way as total cholesterol.
