# Supplementary material for: Sex differences in the association between major cardiovascular risk factors in midlife and dementia: a cohort study using data from the UK Biobank
Source: BMC Med. 2021 May 19;19:110. doi: 10.1186/s12916-021-01980-z (PMC8132382; doi:10.1186/s12916-021-01980-z)
Supplement: Supplementary file 6 — Additional file 6. Multiple adjusted hazard ratios (HRs) and 95% confidence intervals (CI) for the association between risk factors and incident dementia, by socioeconomic status (SES) and sex. [file 12916_2021_1980_MOESM6_ESM.docx]

**Additional file 6: Multiple adjusted hazard ratios** **(HRs) and 95% confidence intervals (CI) for the association between risk factors and incident dementia, by socioeconomic status (SES) and sex.**

| **Risk factors** | **SES subgroups** | **Women HR (95% CI)** | **Men HR (95% CI)** | **RHR (95% CI)** | **P for interaction** |
| --- | --- | --- | --- | --- | --- |
| Systolic blood pressure (per 20mmHg) | Higher SES | 1.12 (1.05, 1.20) | 0.96 (0.90, 1.02) | 1.17 (1.07, 1.29) | 0.03 |
|  | Lower SES | 1.01 (0.93, 1.10) | 1.01 (0.93, 1.10) | 1.00 (0.89, 1.12) |  |
| Diastolic blood pressure (per 10mmHg) | Higher SES | 1.06 (1.00, 1.13) | 0.89 (0.84, 0.94) | 1.20 (1.09, 1.31) | 0.01 |
|  | Lower SES | 0.94 (0.87, 1.02) | 0.99 (0.92, 1.06) | 0.95 (0.85, 1.07) |  |
| Elevated blood pressure vs Normal blood pressure | Higher SES | 0.75 (0.61, 0.92) | 0.85 (0.71, 1.02) | 0.88 (0.67, 1.16) | 0.36 |
|  | Lower SES | 0.89 (0.71, 1.11) | 0.75 (0.59, 0.95) | 1.19 (0.86, 1.64) |  |
| Stage 1 hypertension vs Normal blood pressure | Higher SES | 0.95 (0.84, 1.08) | 0.79 (0.70, 0.89) | 1.20 (1.01, 1.43) | 0.42 |
|  | Lower SES | 0.78 (0.66, 0.92) | 0.81 (0.70, 0.94) | 0.96 (0.77, 1.20) |  |
| Stage 2 hypertension vs Normal blood pressure | Higher SES | 1.08 (0.99, 1.18) | 0.77 (0.71, 0.83) | 1.41 (1.26, 1.58) | 0.24 |
|  | Lower SES | 0.88 (0.79, 0.99) | 0.82 (0.74, 0.90) | 1.08 (0.93, 1.25) |  |
| Former smoker vs Never smoker | Higher SES | 1.06 (0.96, 1.17) | 1.16 (1.07, 1.25) | 0.91 (0.81, 1.04) | 0.70 |
|  | Lower SES | 1.02 (0.90, 1.15) | 1.07 (0.97, 1.18) | 0.95 (0.81, 1.11) |  |
| Current smoker vs Never smoker | Higher SES | 1.24 (0.97, 1.58) | 1.38 (1.15, 1.65) | 0.90 (0.67, 1.22) | 0.07 |
|  | Lower SES | 1.66 (1.38, 1.98) | 1.27 (1.09, 1.50) | 1.30 (1.02, 1.65) |  |
| 1-9 cigarettes per day vs Never smoker | Higher SES | 0.86 (0.46, 1.59) | 1.72 (1.02, 2.90) | 0.50 (0.22, 1.12) | 0.32 |
|  | Lower SES | 1.39 (0.85, 2.26) | 1.63 (1.01, 2.62) | 0.85 (0.43, 1.68) |  |
| 10-19 cigarettes per day vs Never smoker | Higher SES | 1.29 (0.87, 1.93) | 1.65 (1.16, 2.36) | 0.78 (0.46, 1.34) | 0.12 |
|  | Lower SES | 1.43 (1.05, 1.95) | 1.05 (0.75, 1.48) | 1.36 (0.86, 2.15) |  |
| ≥ 20 cigarettes per day vs Never smoker | Higher SES | 1.52 (0.92, 2.53) | 2.33 (1.69, 3.22) | 0.65 (0.36, 1.19) | 0.11 |
|  | Lower SES | 1.82 (1.31, 2.52) | 1.55 (1.20, 2.01) | 1.17 (0.77, 1.78) |  |
| Type 1 diabetes vs No diabetes | Higher SES | 3.55 (1.47, 8.56) | 2.87 (1.37, 6.03) | 1.24 (0.39, 3.91) | 0.68 |
|  | Lower SES | 2.60 (0.84, 8.09) | 2.75 (1.14, 6.60) | 0.95 (0.23, 3.98) |  |
| Type 2 diabetes vs No diabetes | Higher SES | 1.51 (1.20, 1.92) | 1.82 (1.56, 2.12) | 0.83 (0.63, 1.10) | 0.67 |
|  | Lower SES | 1.97 (1.60, 2.43) | 2.00 (1.72, 2.33) | 0.99 (0.76, 1.28) |  |
| Body mass index (per 5kg/m^2^) | Higher SES | 1.00 (0.94, 1.06) | 0.95 (0.88, 1.02) | 1.05 (0.95, 1.15) | 0.70 |
|  | Lower SES | 1.12 (1.05, 1.20) | 1.10 (1.02, 1.18) | 1.02 (0.93, 1.13) |  |
| Waist circumference (per 10 cm) | Higher SES | 1.69 (0.98, 2.91) | 1.53 (0.49, 4.75) | 1.10 (0.31, 3.87) | 0.61 |
|  | Lower SES | 2.17 (1.20, 3.93) | 1.55 (0.58, 4.13) | 1.41 (0.45, 4.42) |  |
| Waist to hip ratio (per 0.1) | Higher SES | 0.86 (0.78, 0.94) | 0.76 (0.70, 0.82) | 1.13 (1.00, 1.28) | 0.50 |
|  | Lower SES | 1.06 (0.94, 1.20) | 0.89 (0.80, 0.98) | 1.20 (1.02, 1.41) |  |
| Waist to height ratio (per 0.1) | Higher SES | 0.96 (0.85, 1.09) | 0.84 (0.75, 0.94) | 1.14 (0.96, 1.35) | 0.64 |
|  | Lower SES | 1.30 (1.14, 1.47) | 1.06 (0.94, 1.19) | 1.22 (1.03, 1.46) |  |
| Underweight vs Healthy weight | Higher SES | 1.03 (0.98, 1.09) | 0.99 (0.94, 1.04) | 1.04 (0.97, 1.12) | 0.90 |
|  | Lower SES | 1.14 (1.08, 1.20) | 1.10 (1.04, 1.16) | 1.03 (0.96, 1.12) |  |
| Overweight vs Healthy weight | Higher SES | 1.13 (1.04, 1.23) | 1.03 (0.94, 1.12) | 1.10 (0.97, 1.24) | 0.44 |
|  | Lower SES | 1.30 (1.18, 1.44) | 1.29 (1.17, 1.42) | 1.01 (0.88, 1.17) |  |
| Obese vs Healthy weight | Higher SES | 1.08 (1.00, 1.17) | 1.04 (0.95, 1.14) | 1.04 (0.92, 1.17) | 0.54 |
|  | Lower SES | 1.30 (1.19, 1.42) | 1.32 (1.21, 1.45) | 0.98 (0.87, 1.11) |  |
| History of stroke vs No history | Higher SES | 2.88 (2.17, 3.83) | 2.37 (1.89, 2.98) | 1.21 (0.84, 1.75) | 0.35 |
|  | Lower SES | 2.34 (1.66, 3.29) | 2.49 (1.96, 3.14) | 0.94 (0.62, 1.42) |  |
| Total cholesterol (per 1 mmol/L) | Higher SES | 1.04 (0.98, 1.11) | 1.02 (0.96, 1.08) | 1.03 (0.94, 1.12) | 0.33 |
|  | Lower SES | 1.03 (0.96, 1.12) | 0.92 (0.86, 1.00) | 1.12 (1.00, 1.25) |  |
| HDL cholesterol (per 1 mmol/L) | Higher SES | 1.02 (0.85, 1.23) | 1.11 (0.91, 1.35) | 0.92 (0.71, 1.21) | 0.28 |
|  | Lower SES | 0.83 (0.65, 1.06) | 1.09 (0.86, 1.39) | 0.76 (0.54, 1.07) |  |
| LDL cholesterol (per 1 mmol/L) | Higher SES | 1.06 (0.98, 1.14) | 1.02 (0.94, 1.10) | 1.04 (0.93, 1.16) | 0.23 |
|  | Lower SES | 1.07 (0.97, 1.18) | 0.91 (0.82, 1.01) | 1.18 (1.03, 1.36) |  |
| Elevated cholesterol vs Normal cholesterol | Higher SES | 1.07 (0.93, 1.23) | 1.04 (0.90, 1.21) | 1.02 (0.84, 1.25) | 0.49 |
|  | Lower SES | 1.16 (0.97, 1.38) | 0.97 (0.79, 1.20) | 1.19 (0.90, 1.56) |  |

HR, hazard ratio; CI, confidence interval; RHR, ratio of hazard ratios; SES, socioeconomic status; HDL, high density lipoprotein; LDL, low density lipoprotein.

The two-level SES was categorised as above or below the UK national median Townsend score (-0.56). N = 335 264 in the higher SES group, and N = 166 339 in the lower SES group.

Hazard ratios were calculated from separate models with different sets of covariate adjustment. All models were adjusted for age and Townsend index. In addition, SBP, diabetes and total cholesterol were adjusted for each other, as well as smoking status, body mass index, lipid lowering drugs and antihypertensive drugs. Same adjustments were made for DBP and AHA hypertension as for SBP. History of stroke and smoking variables were adjusted for socioeconomic status. Body adiposity variables were adjusted for smoking. HDL, LDL cholesterol and elevated cholesterol were adjusted the same way as total cholesterol.
