# Supplementary material for: Sex differences in the association between major cardiovascular risk factors in midlife and dementia: a cohort study using data from the UK Biobank
Source: BMC Med. 2021 May 19;19:110. doi: 10.1186/s12916-021-01980-z (PMC8132382; doi:10.1186/s12916-021-01980-z)
Supplement: Supplementary file 7 — Additional file 7. Unadjusted rates of incident dementia (per 10,000 person years) by sex, and women-to-men difference of rate differences for each risk factor. [file 12916_2021_1980_MOESM7_ESM.docx]

**Additional file 7: Unadjusted rates of incident dementia (per 10 000 person years) by sex, and women-to-men difference of rate differences for each risk factor.**

| **Variables** | **Rates/10 000 person years (95% CI)** | | **Difference of rate differences (95% CI)** |
| --- | --- | --- | --- |
|  | **Women (n=273 262)** | **Men (n=228 964)** |  |
| AHA hypertension categories: |  |  |  |
| Normal | 3.20 (2.77, 3.64) | 8.04 (6.87, 9.20) | – |
| Elevated | 4.48 (3.83, 5.12) | 7.18 (6.22, 8.15) | - 2.12 (- 3.82, - 0.42) |
| Stage 1 hypertension | 4.90 (4.43, 5.38) | 7.05 (6.44, 7.67) | - 2.68 (- 4.15, - 1.22) |
| Stage 2 hypertension | 8.30 (7.80, 8.80) | 9.43 (8.92, 9.95) | - 3.70 (- 5.14, - 2.26) |
| Smoking status: |  |  |  |
| Never smoker | 5.36 (5.03, 5.69) | 6.49 (6.05, 6.93) | – |
| Former smoker | 6.52 (6.01, 7.02) | 10.71 (10.07, 10.36) | 3.07 (2.08, 4.05) |
| Current smoker | 6.73 (5.77, 7.70) | 8.55 (7.54, 9.57) | 0.69 (- 0.82, 2.19) |
| Smoking intensity (average number of cigarettes smoked daily): | | | |
| Never | 5.36 (5.03, 5.69) | 6.49 (6.05, 6.93) | – |
| 1-9 cigarettes | 5.13 (3.16, 7.11) | 9.81 (6.36, 13.27) | 3.55 (- 0.47, 7.56) |
| 10-19 cigarettes | 6.62 (5.00, 8.24) | 8.08 (6.08, 10.07) | -0.32 (- 2.30, 2.95) |
| ≥20 cigarettes | 8.06 (5.85, 10.27) | 11.00 (8.79, 13.21) | 1.81 (- 1.37, 4.98) |
| Diabetes: |  |  |  |
| No diabetes | 5.47 (5.21, 5.73) | 7.36 (7.02, 7.71) | – |
| Type 1 | 16.00 (6.08, 25.91) | 18.59 (8.49, 28.70) | 0.70 (- 13.46, 14.87) |
| Type 2 | 16.19 (13.83, 18.54) | 22.51 (20.24, 24.78) | 4.43 (1.12, 7.73) |
| Body mass index: |  |  |  |
| Healthy weight (18.5 – 24.9) | 5.12 (4.72, 5.52) | 8.88 (8.16, 9.61) | – |
| Underweight (<18.5) | 10.13 (6.08, 14.18) | 12.43 (3.22, 21.63) | - 1.46 (- 11.55, 8.63) |
| Overweight (25.0 – 29.9) | 5.85 (5.41, 6.29) | 7.72 (7.24, 8.20) | - 1.89 (- 2.95, - 0.84) |
| Obese (30.0 and above) | 7.03 (6.44, 7.63) | 9.26 (8.54, 9.99) | - 1.54 (- 2.79, - 0.28) |
| Stroke: |  |  |  |
| None | 5.67 (5.41, 5.93) | 7.94 (7.60, 8.29) | – |
| History of stroke | 24.40 (19.21, 29.58) | 32.47 (27.38, 37.57) | 5.80 (- 1.48, 13.09) |
| Townsend deprivation thirds: |  |  |  |
| High | 5.32 (4.96, 5.67) | 7.50 (7.04, 7.97) | – |
| Middle | 5.69 (5.21, 6.17) | 8.13 (7.49, 8.77) | 0.25 (- 0.74, 1.24) |
| Low | 7.74 (7.03, 8.44) | 11.16 (10.25, 12.07) | 1.24 (- 0.05, 2.53) |
| Lipids: |  |  |  |
| Normal cholesterol | 5.49 (5.16, 5.83) | 8.97 (8.54, 9.41) | – |
| Elevated cholesterol | 6.45 (6.01, 6.88) | 7.13 (6.55, 7.72) | -2.79 (-3.71, - 1.88) |

AHA, American Heart Association.
