# Supplementary material for: Sex differences in the association between major cardiovascular risk factors in midlife and dementia: a cohort study using data from the UK Biobank
Source: BMC Med. 2021 May 19;19:110. doi: 10.1186/s12916-021-01980-z (PMC8132382; doi:10.1186/s12916-021-01980-z)
Supplement: Supplementary file 8 — Additional file 8. Kaplan-Meier survival curves for death and dementia, by sex. [file 12916_2021_1980_MOESM8_ESM.docx]

**Additional file 8: Kaplan-Meier survival curves for death and dementia, by sex.**

A: Outcome: Death


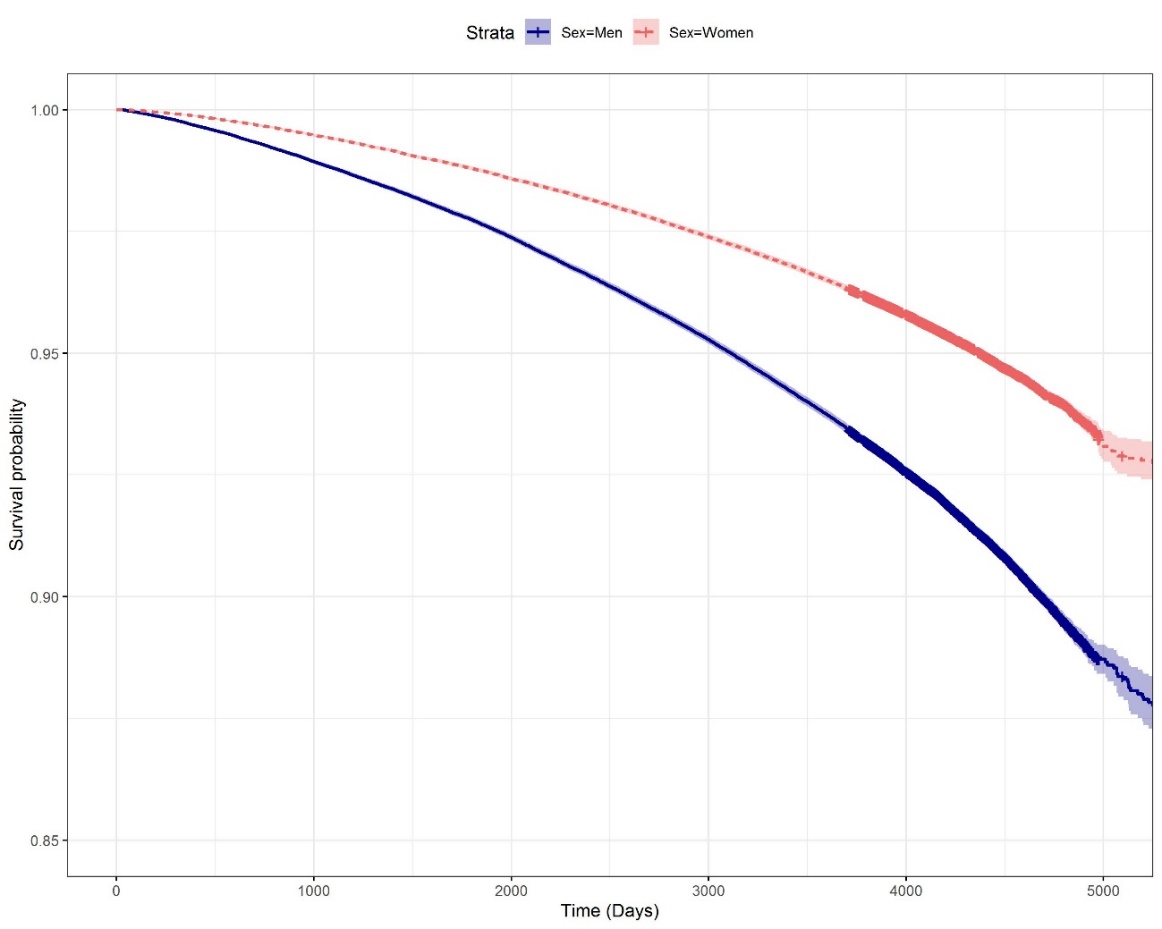


| At risk time (days) | 0 | 1000 | 2000 | 3000 | 4000 | End of follow-up  30/11/2020 |
| --- | --- | --- | --- | --- | --- | --- |
| Women | 273,262 | 271,831 | 269,388 | 266,125 | 215,638 | 213,505 |
| Cumulative deaths |  | 1431 | 3874 | 7137 | 11,377 | 13,510 |
|  |  |  |  |  |  |  |
| Men | 228,964 | 226,522 | 222,967 | 218,154 | 174,365 | 171,466 |
| Cumulative deaths |  | 2442 | 5997 | 10,810 | 16,876 | 19,775 |

B: Outcome: Dementia


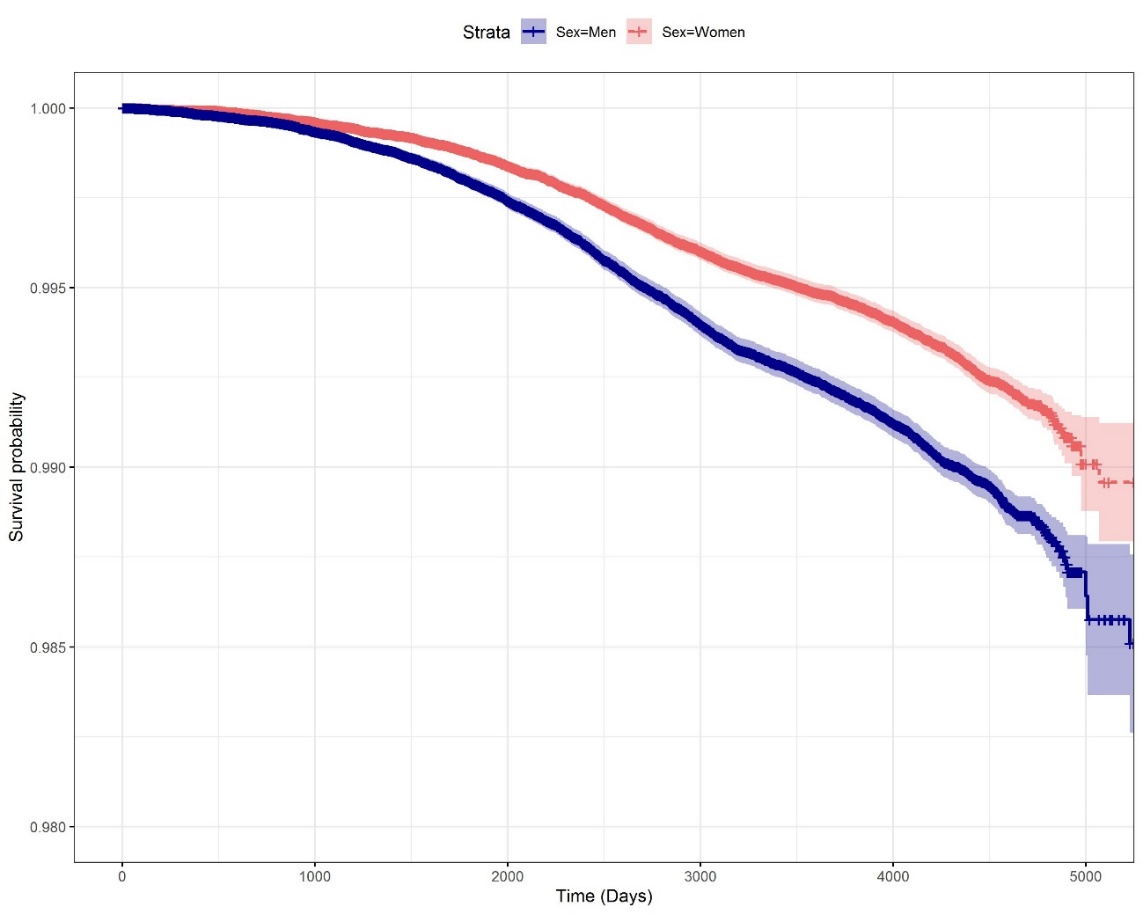


| At risk time (days) | 0 | 1000 | 2000 | 3000 | 4000 | End of follow-up  30/11/2020 |
| --- | --- | --- | --- | --- | --- | --- |
| Women | 273,262 | 271,739 | 269,038 | 265,345 | 214,879 | 214,590 |
| Cumulative Dementia events |  | 108 | 437 | 1073 | 1577 | 1866 |
|  |  |  |  |  |  |  |
| Men | 228,964 | 226,394 | 222,546 | 217,311 | 173,658 | 173,377 |
| Cumulative Dementia events |  | 149 | 583 | 1340 | 1921 | 2202 |
