# Supplementary material for: Sex differences in the association between major cardiovascular risk factors in midlife and dementia: a cohort study using data from the UK Biobank
Source: BMC Med. 2021 May 19;19:110. doi: 10.1186/s12916-021-01980-z (PMC8132382; doi:10.1186/s12916-021-01980-z)
Supplement: Supplementary file 9 — Additional file 9. Multiple-adjusted sub-distribution hazard ratios (SHR) and ratio of the sub-distribution hazard ratios (women-to-men) for risk factors for dementia from competing risks analysis. [file 12916_2021_1980_MOESM9_ESM.docx]

**Additional file 9: Multiple-adjusted sub-distribution hazard ratios (SHR) and ratio of the sub-distribution hazard ratios (women-to-men) for risk factors for dementia from competing risks analysis**

| **Risk factors** | **Women**  **SHR (95% CI)** | **Men**  **SHR (95% CI)** | **Women to men**  **RSHR (95% CI)** |
| --- | --- | --- | --- |
| Systolic blood pressure (per 20mmHg) | 1.08 (1.02, 1.14) | 0.98 (0.93, 1.03) | 1.10 (1.02, 1.19) |
| Diastolic blood pressure (per 10mmHg) | 1.01 (0.96, 1.07) | 0.93 (0.89, 0.98) | 1.09 (1.01, 1.17) |
| AHA categories: |  |  |  |
| Elevated blood pressure vs Normal blood pressure | 0.81 (0.66, 1.00) | 0.83 (0.67, 1.03) | 0.98 (0.73, 1.32) |
| Stage 1 hypertension vs Normal blood pressure | 0.89 (0.74, 1.06) | 0.82 (0.68, 0.99) | 1.08 (0.83, 1.39) |
| Stage 2 hypertension vs Normal blood pressure | 1.00 (0.85, 1.18) | 0.81 (0.68, 0.96) | 1.24 (0.98, 1.57) |
| Former smoker vs Never smoker | 1.03 (0.93, 1.13) | 1.10 (1.00, 1.20) | 0.93 (0.82, 1.07) |
| Current smoker vs Never smoker | 1.40 (1.19, 1.67) | 1.23 (1.06, 1.43) | 1.13 (0.91, 1.41) |
| Smoking intensity: |  |  |  |
| 1-9 cigarettes per day vs Never smoker | 1.07 (0.73, 1.59) | 1.61 (1.12, 2.31) | 0.68 (0.39, 1.13) |
| 10-19 cigarettes per day vs Never smoker | 1.29 (1.00, 1.67) | 1.19 (0.92, 1.55) | 1.08 (0.75, 1.56) |
| ≥ 20 cigarettes per day vs Never smoker | 1.56 (1.17, 2.08) | 1.62 (1.30, 2.02) | 0.96 (0.67, 1.38) |
| Type 1 diabetes vs No diabetes | 2.73 (1.35, 5.52) | 2.63 (1.49, 4.64) | 1.04 (0.42, 2.57) |
| Type 2 diabetes vs No diabetes | 1.69 (1.42, 2.02) | 1.83 (1.61, 2.09) | 0.92 (0.74, 1.15) |
| Body mass index (per 5kg/m^2^) | 1.05 (1.00, 1.10) | 1.01 (0.95, 1.07) | 1.04 (0.96, 1.12) |
| Waist circumference (per 10 cm) | 1.07 (1.03, 1.12) | 1.03 (0.98, 1.07) | 1.04 (0.98, 1.11) |
| Waist to hip ratio (per 0.1) | 1.18 (1.11, 1.26) | 1.10 (1.03, 1.18) | 1.07 (0.98, 1.18) |
| Waist to height ratio (per 0.1) | 1.16 (1.09, 1.24) | 1.13 (1.06, 1.22) | 1.03 (0.93, 1.13) |
| BMI categories: |  |  |  |
| Underweight vs Healthy weight | 1.79 (1.19, 2.69) | 1.24 (0.58, 2.63) | 1.44 (0.61, 3.40) |
| Overweight vs Healthy weight | 0.92 (0.82, 1.02) | 0.81 (0.73, 0.89) | 1.14 (0.98, 1.32) |
| Obese vs Healthy weight | 1.06 (0.94, 1.19) | 0.90 (0.81, 1.02) | 1.17 (0.99, 1.38) |
| History of stroke vs No history | 2.50 (2.00, 3.11) | 2.27 (1.92, 2.68) | 1.10 (0.86, 1.15) |
| Middle SES vs High SES | 1.12 (1.00, 1.26) | 1.15 (1.04, 1.28) | 0.97 (0.83, 1.13) |
| Low SES vs High SES | 1.64 (1.46, 1.85) | 1.52 (1.36, 1.70) | 1.08 (0.92, 1.27) |
| Total cholesterol (per 1 mmol/L) | 1.04 (0.99, 1.09) | 0.99 (0.94, 1.04) | 1.06 (0.98, 1.13) |
| HDL cholesterol (per 1 mmol/L) | 0.95 (0.82, 1.11) | 1.11 (0.94, 1.30) | 0.86 (0.69, 1.07) |
| LDL cholesterol (per 1 mmol/L) | 1.06 (1.00, 1.13) | 0.98 (0.92, 1.05) | 1.08 (0.99, 1.19) |
| Elevated cholesterol vs Normal cholesterol | 1.10 (0.99, 1.23) | 1.03 (0.91, 1.16) | 1.08 (0.91, 1.27) |

AHA, American Heart Association; BMI, body mass index; SES, socioeconomic status.
